# Functional and omics-based rationale for the induction of BRCAness by androgen receptor pathway inhibitors to sensitize prostate cancer to PARP inhibition, regardless of HRR status

**DOI:** 10.1038/s41416-026-03518-7

**Published:** 2026-07-07

**Authors:** Mohamed E. Elsesy, Ayham Moustafa, Su-Jung Oh-Hohenhorst, Christian Müller, Malik Alawi, Thomas Mair, Bente Siebels, Zifan Hu, Jan Hahn, Susanne Burdak-Rothkamm, Derya Tilki, Hartmut Schlüter, Cordula Petersen, Tobias Maurer, Gunhild von Amsberg, Carsten Bokemeyer, Kai Rothkamm, Wael Y. Mansour

**Affiliations:** 1 Department of Radiotherapy and Radiooncology, University Medical Center Hamburg–Eppendorf, Hamburg, Germany; 2 Pharmacology and Experimental Oncology Unit, Cancer Biology Department, National Cancer Institute, Cairo University, Cairo, Egypt; 3 Section for Mass Spectrometry and Proteomics, Center for Diagnostics, University Medical Center Hamburg-Eppendorf, Hamburg, Germany; 4 Martini-Klinik Prostate Cancer Center; University Medical Center Hamburg-Eppendorf, Hamburg, Germany; 5 Department of Urology, University Hospital Hamburg-Eppendorf, Hamburg, Germany; 6 Bioinformatics Core, University Medical Center Hamburg-Eppendorf, Hamburg, Germany; 7 Mildred Scheel Cancer Career Center HaTriCS4, University Medical Center Hamburg-Eppendorf, Hamburg, Germany; 8 Department of Urology, Koc University Hospital, Istanbul, Türkiye; 9 Department of Oncology, Hematology and Bone Marrow Transplantation with Section of Pneumology; University Medical Center Hamburg–Eppendorf, Hamburg, Germany

**Keywords:** Cancer, Cancer therapy

## Abstract

**Background:**

Two PARP inhibitors (PARPis), olaparib and talazoparib, have been approved in combination with androgen receptor pathway inhibitors (ARPIs) for metastatic castration-resistant prostate cancer (mCRPC) in Europe, regardless of homologous-recombination repair (HRR) status. However, the mechanism in HRR-negative patients remains unclear.

**Methods:**

We assessed PARP inhibitors (PARPis; olaparib, talazoparib) alone and with ARPIs (abiraterone, enzalutamide, apalutamide) in prostate cancer cell lines, metastatic patient-derived organoids (mPDOs), and LN-metastatic tissue slice cultures. HRDetect and multi-omics analyses were used to investigate response mechanisms.

**Results:**

PARPi-ARPI combinations significantly reduced survival in LNCaP, DU145, and 22RV1 cells and produced greater cytotoxicity than either agent alone in all models. In three metastatic mPDOs, olaparib alone had no effect, whereas talazoparib reduced survival in one mPDO and ARPIs reduced survival in two. In all tested models, PARPi-ARPI combinations produced greater cytotoxicity than either agent alone. None of the mPDOs showed BRCAness by HRDetect. ARPIs downregulated RAD51 and induced a BRCAness-like state, with reduced RAD51 foci formation at DNA double-strand break sites and impaired repair capacity, thereby sensitizing cells to PARPis. In LN-metastatic tissue slice cultures, combination therapy reduced double-strand break repair capacity in 67% of samples. Proteomic analysis showed that olaparib suppressed prometastatic pathways, including epithelial-mesenchymal transition and angiogenesis.

**Conclusion:**

ARPIs induce a BRCAness-like phenotype through RAD51 downregulation and enhance PARPi sensitivity in metastatic prostate cancer models, supporting combined ARPI-PARPi therapy.

## Background

Despite advancements in prostate cancer (PCa) treatments that have improved survival rates, PCa remains the second leading cause of cancer death in men in Western countries, with a poor prognosis for those with metastatic disease, especially metastatic castration-resistant prostate cancer (mCRPC), which has a 5-year survival rate of only 30% [1]. Although androgen deprivation therapy (ADT) is the cornerstone treatment for metastatic PCa (mPCa), it eventually results in therapeutic failure due to the development of resistance [2]. Novel androgen receptor pathway inhibitors (ARPis), such as abiraterone, apalutamide, and enzalutamide, improve survival, but these responses are often short-lived because of the development of resistance [2, 3]. This highlights the unmet clinical need for new treatment strategies to provide longer-lasting responses for mPCa patients.

Poly (ADP‒ribose) polymerase (PARP) inhibitors (PARPis) are revolutionizing cancer treatment by targeting the PARP active site, competing with NAD+ for binding and exploiting synthetic lethality (SL) in tumors with ‘’BRCAness”, which definedby functional deficiencies in homologous recombination repair (HRR), classically due to BRCA1/2 mutations [4] but also arising from broader HRR gene defects. Approximately 30% of mCRPCs exhibit alterations across DDR genes, but PARPi efficacy is primarily limited to the ~ 5-8% with BRCA1/2 mutations. Phase I/III trials (PROfound, TRITON2) demonstrate significant progression-free survival (PFS) benefit in BRCA1/2-mutant mCRPC (HR 034-0.49), with limited activity in other DDR alterations [5, 6], highlighting the need for strategies expanding PARPi utility to HRR-proficient tumors. Clinical trials have shown that PARPis are effective for these patients, especially those with harmful BRCA1/2 mutations, as second- or third-line treatments

Phase II/III trials (PROfound, TRITON2) demonstrate significant PFS benefit in BRCA1/2-mutant mCRPC (HR 0.34–0.49), with limited activity in other DDR alterations, highlighting the need for strategies expanding PARPi utility to HRR-proficient tumors.

Preclinical data have suggested cross-talk between the androgen receptor (AR) signal transduction pathway and PARP [7–9]. On the one hand, PARPis may influence AR transcriptional activity, potentially augmenting the antitumor effects of ARPIs. On the other hand, AR regulates the expression of several DNA repair genes involved in DNA damage sensing, nonhomologous end joining (NHEJ), HRR, mismatch repair (MMR), and the Fanconi pathway [10]. As a result, ARPIs may reduce the expression of these genes, thereby improving the efficacy of PARPis. Several clinical trials have contributed to the growing evidence for combination therapies in mCRPC, highlighting the effectiveness of integrating PARPis with ARPIs to improve patient outcomes. Two phase III trials, namely, PROpel and TALAPRO-2, evaluated the efficacy of combined PARPi and ARPI therapy in an all-comer population. The PROpel trial demonstrated that combining olaparib with abiraterone/prednisone significantly prolonged progression-free survival (PFS) compared with ARPI monotherapy in the first-line treatment of mCRPC. [11]. These findings were further confirmed in Cohort 1 of the TALAPRO-2 study, which evaluated the combination of the PARPi talazoparib with the ARPI enzalutamide in HRR-unselected mCRPC patients [12]. Together, the findings from these trials led to several approvals for various combination regimens of PARPis and ARPIs for mCRPC patients. For example, the FDA approved olaparib with abiraterone and prednisone/prednisolone for BRCA-mutated mCRPCa, whereas the European Commission approved this combination for mCRPC regardless of HRR status.

Despite the success of this combination as an alternative treatment for PCa, the exact biological mechanism is not fully understood. Additionally, while BRCA1/2 carriers showed the greatest benefit in subgroup analyses of the PROpel and TALAPRO2 trials, patients without pathological HRR alterations also experienced significant advantages. To address these findings, we investigated the mechanistic effects of combining different PARPis with various ARPIs on androgen-sensitive and castration-resistant human PCa cell lines, ex vivo tissue slice cultures (TSCs), and patient-derived organoids (PDOs) from metastatic PCa patients with or without castration-resistant features. Our findings demonstrated that combining ARPI and PARPi was more toxic across all the models tested, regardless of whether the treatment was administered simultaneously or sequentially. Mechanistically, we provide solid functional and omics-based evidence that ARPI treatment induces a BRCAness phenotype, which enhances sensitivity to PARPis. In summary, we present a mechanistic basis for combining ARPIs with PARPis, irrespective of treatment order, as a therapeutic strategy for mPCa.

## Materials and methods

### Cell culture, drugs, and X-irradiation

LNCaP (RRID:CVCL_0395), DU145 (RRID:CVCL_0105), and 22-Rv1 (RRID:CVCL_1045) cells were maintained in DMEM (Gibco, Invitrogen, cat # 41966161) supplemented with 10% fetal calf serum, 100 U/ml penicillin, and 100 µg/ml streptomycin at 37 °C under 10% CO2. All the cell lines were provided by collaboration partners or purchased from the American Type Culture Collection (ATCC), tested and confirmed to be free of mycoplasma contamination. Additionally, all cell lines were authenticated by short tandem repeat (STR) profiling to confirm their identity and genetic consistency. Olaparib (AZD2281), enzalutamide (MDV3100) and talazoparib (BMN 673) were obtained from Selleckchem (Cat. No. S1060, S1250 and S7048, respectively). Abiraterone acetate and apalutamide were kindly provided by Janssen Cilag GmbH (Neuss, Germany) as a part of a nonclinical investigator-initiated study (ARN-I-15-DEU-004). Irradiation was performed as previously described at 200 kV and 15 mA with a 0.5 mm Cu filter and a dose rate of 0.8 Gy/min [13].

### Colony-forming assay (CFA)

Cellular survival was determined via a colony formation assay as previously described [13]. Surviving fractions (SFs) were calculated by normalizing the colony counts to the plating efficiency of the untreated control.

### RNA isolation, RNA sequencing and transcriptomic analysis

Total RNA was isolated from untreated and treated cells via an RNeasy Mini Kit (Qiagen, Hilden, Germany, Cat # 74904) and subsequently sent to Novogene (Munich, Germany) for RNA-SEQ library preparation and sequencing. Data analysis was performed by Novogene and further validated in the Bioinformatics core facility at UKE.

### Homologous recombination deficiency (HRD) detection in whole-genome sequencing samples

Organoids were dissociated, and genomic DNA was isolated using a DNeasy mini Kit (Qiagen, Hilden, Germany, Cat #69504) according to the manufacturer’s protocol, then sent to Novogene (Munich, Germany) for whole-genome sequencing (WGS) library preparation and sequencing. Paired-end sequencing (PE150) was performed on a NovaSeq X Plus system to achieve 30× coverage ( ~ 90 Gb per line). To estimate the probability of HRD, the HRDetect workflow implemented in the R package signature.tools.lib [14] was applied to each sample. Therefore, the proportion of small insertions/deletions at microhomology and single base substitution signatures (SBS) based on COSMIC signatures provided by Degasperi et al. was calculated using the filtered somatic variants [15]. Structural somatic variants were used to estimate known rearrangement signatures (RS). Loss of heterozygosity was calculated from CNVs. The function HRDetect_pipeline was applied to estimate the BRCAness score reflecting the HRD probability based on RS3/RS5, SBS3/SBS8, deletion microhomology, and loss of heterozygosity. Samples with a BRCAness score ≥ 0.7 were classified as HRD. Raw reads underwent quality control, adapter trimming, filtering, and base correction using fastp [16], followed by alignment to GRCh38 (GCA_000001405.15) via bwa [17]. Samtools [18] was used for sorting and duplicate marking. DeepSomatic [19] in tumor-only mode with “--use_default_pon_filtering=true” identified somatic variants, retaining those with depth ≥10 and QUAL ≥ 30 for annotation via Ensembl VEP [20]. Structural variants were called using Manta [21], and microsatellite instability (MSI) was assessed with MSIsensor2 (https://github.com/niu-lab/msisensor2) with “models_hg38”. MSI ≥ 20% was considered unstable. Copy number variation (CNV), purity, and ploidy were analyzed using ezASCAT (https://github.com/CompEpigen/ezASCAT) [22] in tumor-only mode. HRDetect_pipeline (R/signature.tools.lib [14]) estimated: (i) deletion microhomology (from SVs), (ii) SBS3/SBS8 (from SNPs), (iii) RS3/RS5 (from short indels), (iv) loss of heterozygosity (from CNVs), and (v) HRDetect scores.

### Western blotting

Whole-cell lysates were analyzed by western blotting as described previously [23]. Antibodies used: Rabbit anti-RAD51 antibody (RRID:AB_2238184), rabbit polyclonal anti-53BP1 (RRID:AB_10001695), rabbit monoclonal anti-H2AX (RRID:AB_10860771), a mouse anti-HSC70 antibody (RRID:AB_627761), goat anti-mouse IgG-AlexaFluor 594 (RRID:AB_141372) and goat anti-rabbit IgG-AlexaFluor 488 (RRID:AB_143165).

### Immunofluorescence

Immunofluorescence was performed as previously described [13]. DSBs were quantified via ImageJ (RRID:SCR_003070) with DAPI-based image masks and normalized to single-nucleus values [24]. The primary antibodies used included mouse monoclonal anti-phospho-S139-H2AX (RRID:AB_2755003), rabbit polyclonal anti-53BP1 (RRID:AB_10001695) and rabbit polyclonal anti-RAD51 (RRID:AB_2238184) antibodies.

### Patient sample collection

Fresh PCa tissue samples were collected from patients who underwent either lymph node dissection via prostate-specific membrane antigen (PSMA) radioguided surgery (19 biopsies from 12 PCa patients) or bone metastatic resection (*n* = 3) at the Martini-Klinik, Prostate Cancer Center in Hamburg, Germany, between 2019 and 2022. Immediately following surgical resection, the collected biopsies were placed in culture media and promptly transported to the laboratory. The pseudonymized samples were processed within 30 min of resection. The study was approved by the local ethics committee [Ethik-Kommission der Ärztekammer Hamburg], under project number PV7007. All procedures adhered to the Declaration of Helsinki, and written informed consent was obtained from all participants.

### Ex vivo tissue slice culture

Tissue slice cultures were prepared following our previously described method [24]. Proliferation and oxygenation were routinely assessed as previously described [24].

### Tissue processing and organoid culture generation

Organoid cultures were extracted from metastatic PCa samples following our previously published protocol [25]. Organoids were routinely passaged every 4 to 6 weeks. During passaging, the BME droplets were disrupted mechanically via a P1000 pipette tip and gently treated with TrypLE Express supplemented with 10 μM ROCK inhibitor at 37 °C for up to 5 min. The resulting mixture of single cells and small clumps was washed and replated via the same culture method.

### Histology and imaging

Tissue slice cultures and organoids were fixed for 30 min with 4% PFA (Merck) followed by incubation with 25% sucrose for 2 h. Samples were then embedded and frozen in TissueTek (Sakura Finetek, Alphen aan den Rijin, Netherlands). Thereafter, a Cryo Star NX70 Microtome (Thermo Scientific, Cat# 15360755) was used to generate sections of 5 µm/section on glass slides (Superfrost, Thermo Fisher Scientific, Cat# 10149870). Immunohistochemistry was performed using antibodies against AMACR (1:250 dilution) (RRID:AB_2789895) or Rabbit anti-RAD51 (1:200 dilution) (RRID:AB_2238184). Following Hematoxylin and Eosin (H&E) staining (Roth for hematoxylin, Medite for eosin), digital histological images were examined for tumor cell content by an experienced pathologist. Only slices with ≥70% viable tumor cellularity were included in downstream experiments. All the images were processed with ZEISS Axio Scan Z.1 Slide scanner and netScope viewer. RAD51-positive cells were quantified using the open-source software platform QuPath (version 0.6.4) [26].

### 3D colony formation assay

Organoids were first collected and sheared into single cells before being mixed in cold BME and plated at 4000 cells per dome. Upon complete gelation, different concentrations of ARPIs and/or PARPi were added in triplicate to 500 μl of complete organoid medium. For the sequential treatment regimen, domes were incubated for 24 h with ARPI before PARPi was added. After 3–4 weeks, the organoids encapsulated in domes were stained with 0.5 mg·mL−1 3-(4,5-dimethylthiazolyl-2)-2,5-diphenyltetrazolium bromide (MTT) for 1.5 h. The intact, MTT-stained organoids were then liberated using CultrexTM Organoid Harvesting Solution (R&D Systems. Cat# 3700-100-01) and photographed via REBEL microscopy (RRID:SCR_026523). All the photos were analyzed via ImageJ (RRID:SCR_003070), and SFs were calculated via normalization to the plating efficiency of the untreated control. DMSO was used as a control at the same concentration.

### Differential quantitative proteome analysis

Protein extraction was performed by incubating PDO pellets in 100 mM triethyl ammonium bicarbonate with 1% w/v sodium deoxycholate, followed by boiling at 95 °C for 5 min and sonication. Protein concentration was measured using the Pierce BCA assay (Thermo Fisher), and samples were diluted to 20 μg in 50 µl buffer. Proteins were reduced with 10 mM DTT at 56 °C for 30 min, and alkylated with 20 mM iodoacetamide at 37°C for 30 min in the dark. For each 10 µg of protein, 1 µL of carboxylate-modified magnetic beads (GE Healthcare Sera-Mag™, Chicago, USA) in a 1:1 hydrophilic/hydrophobic mix (methanol/LC‒MS grade water) was used. The concentration of acetonitrile (ACN) in all the samples was increased to 70% following the single-pot, solid-phase enhanced sample preparation (SP3)-protocol workflow. After shaking the samples at 1400 rpm for 18 min at room temperature, the tubes were placed on a magnetic rack to separate the beads from the solution. The bead-free supernatant was discarded, and the magnetic beads were washed with 100% ACN and then with 70% ethanol. 50 mM Ammonium bicarbonate was added and proteins were digested with trypsin (1:100, Promega) at 37°C overnight at 1400 rpm. To allow the generated tryptic peptides to bind to the beads, 95% ACN was added, and the samples were shaken at 1400 rpm for 10 min at room temperature. The bead-free supernatant was removed after the tubes were placed on the magnetic rack. The beads carrying the digested peptides were washed two times with 100% ACN. Elution was performed with 2% DMSO in 1% formic acid. The supernatant was dried in a vacuum centrifuge and stored at -80°C until further use. The tryptic peptides of each sample were resuspended in 0.1% formic acid to a final concentration of 1 μg/μl. A volume of 1 μl was injected into a nano-UHPLC system (Dionex Ultimate 3000 UHPLC system, Thermo Fisher) via an autosampler. Chromatographic separation of peptides was performed via a two-buffer system (buffer A: 0.1% FA in H2O; buffer B: 0.1% FA in ACN). For online desalting and purification, a peptide trap (100 µm × 20 mm, 100 Å pore size, 5 µm particle size, C18, Nano Viper, Thermo Fisher) was attached to the UHPLC, followed by a 25 cm C18 reversed-phase column (75 µm × 250 mm, 130 Å pore size, 1.7 µm particle size, peptide BEH C18, nanoEase, Waters). Peptides were separated via the 80 min method with increasing ACN concentration linearly from 2% to 30% ACN over 60 min. MS/MS measurements were performed on a quadrupole-ion-trap-orbitrap MS (Orbitrap Fusion, Thermo Fisher). Eluting peptides were ionized via a nanoelectrospray ionization source (nano-ESI) with a spray voltage of 1,800 V and analyzed in data-independent acquisition mode. For each MS1 scan, ions were accumulated for a maximum of 120 milliseconds or until a charge density of 2 × 10^5^ ions (AGC Target) was reached. Fourier transformation-based mass analysis of the data from the Orbitrap mass analyzer was performed, covering a mass range of m/z 400–1,300 with a resolution of 120,000 at m/z = 200. Peptides with charge states between 2+ and 5+ above an intensity threshold of 1,000 were isolated within a m/z 1.6 isolation window in top speed mode for 3 s from each precursor scan and fragmented with a normalized collision energy of 30% via higher-energy collisional dissociation. MS2 scanning was performed via an ion trap mass analyzer at a rapid scan rate, covering a mass range starting at m/z 120 and accumulating for 60 ms or to an AGC target of 1 × 105. Already, fragmented peptides were excluded for 30 s.

### Statistical analysis and data visualization

All data were analyzed using GraphPad prism 10.0.0 ((RRID:SCR_002798) and R studio (version 3.6.3), and presented as the mean values  ±  standard error of the mean unless stated otherwise. Group comparisons were made using unpaired Mann-Whitney tests unless otherwise indicated. The choice of non‑parametric test addressed potential deviations from normality. Variation and sample size are reported in figure legends. Variance between groups was comparable, as judged from similar dispersion in replicate measurements. Statistical significance was set at *P*  <  0.05 (two-sided). Cell lines and PDO/TSC experiments: sample size for cell lines, TSCs and PDOs was based on feasibility and prior work. At least three independent biological replicates with technical triplicates per condition. All image‑based readouts such as foci counting, RAD51 IHC, QuPath analyses, were performed in a manner blinded to treatment conditions by automated quantification or pseudonymized sample IDs.

DIA-MS raw files were analyzed with DIA-NN 1.8.1 (RRID:SCR_022865) [27] against the reviewed human SwissProt database (June 2021; 20,386 entries). Carbamidomethylation of cysteines was set as a fixed modification, while methionine oxidation, N-terminal pyroglutamate formation at glutamine, and N-terminal protein acetylation were set as variable modifications. Up to two missing tryptic cleavages were allowed, and peptides of 7-30 amino acids were considered. LC‒MS data were processed in Perseus (RRID:SCR_015753) [28]. Protein abundances were log2-transformed and median-normalized per column to correct the injection-related variation. Gene enrichment analysis was performed with Enrichr [29–31] (RRID:SCR_001575), and bubble charts of enriched terms were generated in R (RRID:SCR_001905). Bar charts of RAD51 abundances and PCA plots were created with GraphPad Prism. Biological duplicates of samples were used in all measurements for proteomics unless stated otherwise.

## Results

### The combination of abiraterone and olaparib outperforms individual treatments in PCa cells

**Fig. 1:**
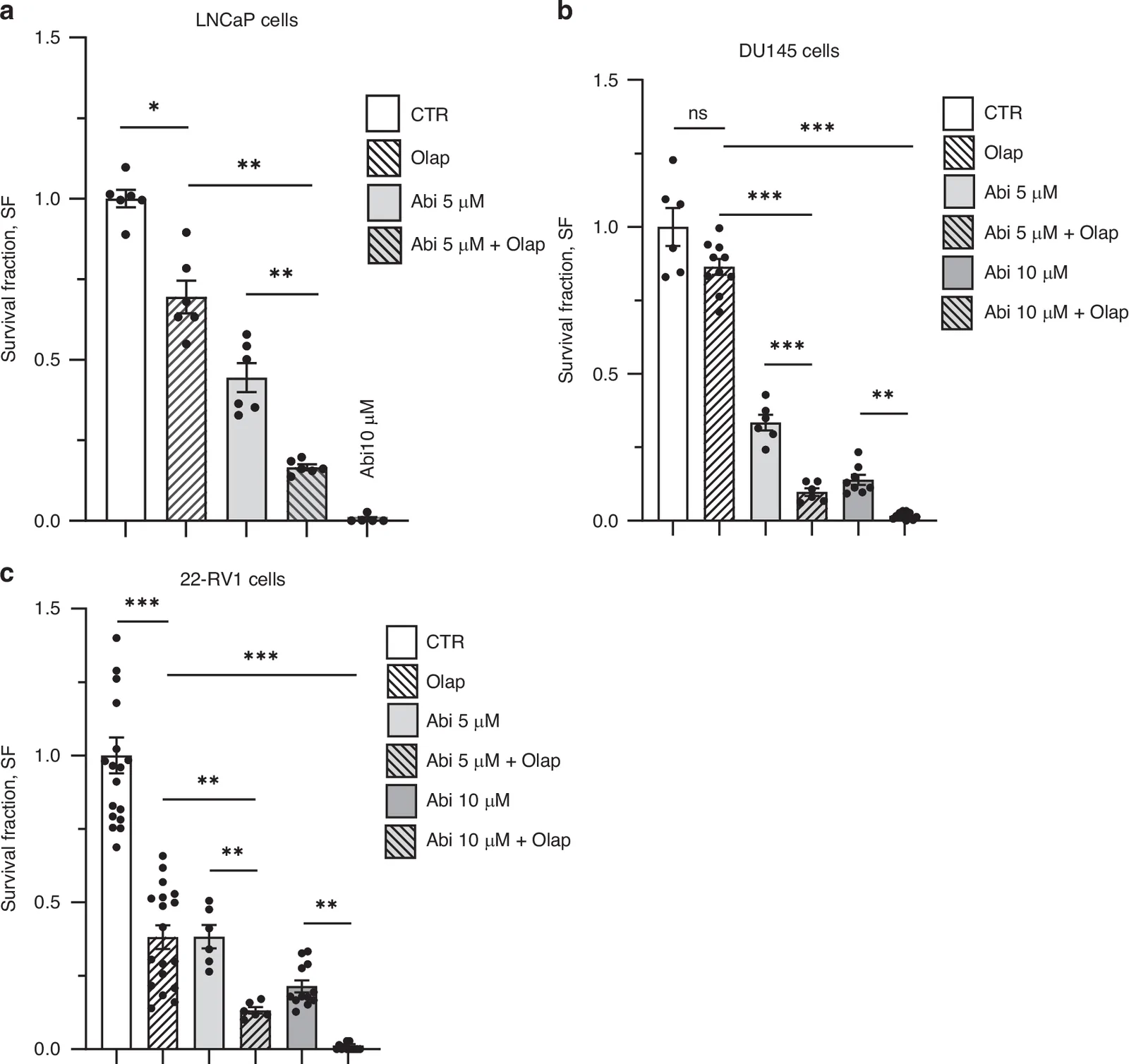
Compared with individual treatments, combined treatment with olaparib and abiraterone has a stronger cytotoxic effect. **a** LNCaP, **b** DU145, and **c** 22RV1 cells were treated with DMSO (CTR), 1 µM olaparib (Olap), abiraterone (Abi) at the indicated concentrations, or the combination of both (Abi+Olap). Cell survival was assessed via a colony formation assay. The data are presented as the means ± SEMs from three independent experiments. Statistical significance was determined with *p* values of * < 0.05, ** < 0.01, and *** < 0.001, ns: not significant.

Initially, we evaluated the effectiveness of combining abiraterone and olaparib in three PCa cell lines with different androgen sensitivity profiles: LNCaP (androgen-sensitive), DU145 (castration-resistant and AR-negative), and 22Rv1 (castration-resistant, AR-positive and splice variant AR-positive). Two abiraterone concentrations were used for all the cell lines: the IC50 (5 µM) determined for LNCaP cells (as previously indicated [13], Supplementary Fig. S1) and a double the IC50 dose (10 µM). Our results revealed varying cellular responses to olaparib among the cell lines tested. While olaparib had no effect on cell survival in LNCaP (Fig. 1a) or DU145 cells (Fig. 1b), it expectedly reduced cell survival by more than 50% in BRCA2-mutated 22RV1 cells (Fig. 1C) [32]. As expected, 5 µM abiraterone reduced LNCaP cell viability by approximately 50%, and at 10 µM, abiraterone fully inhibited LNCaP cell growth (Fig. 1a). Surprisingly, abiraterone induced dose-dependent growth inhibition in both CRPC models DU145 (AR-negative) and 22RV1 (Fig. 1b, c). The robust effect in DU145 cells was particularly striking, consistently observed across ≥3 independent experiments (*n* = 3 biological replicates each, in triplicate). Although low-level AR expression has been reported in DU145 cells [33], this cannot account for the pronounced cytotoxicity. Clinically, abiraterone improves PFS/OS across CRPC patients regardless of AR status (COU-AA-301/302; [34], implicating AR-independent mechanisms such as ER stress, autophagy, CREB1 phosphorylation, and mitochondrial dysfunction [35]. Notably, combining abiraterone with olaparib significantly reduced cell survival across all tested cell lines, demonstrating a stronger effect than either treatment alone. These results validate the effectiveness of combining olaparib with the ARPI abiraterone for the treatment of PCa.

### Abiraterone treatment reduces HRR gene expression and induces HR defects

**Fig. 2:**
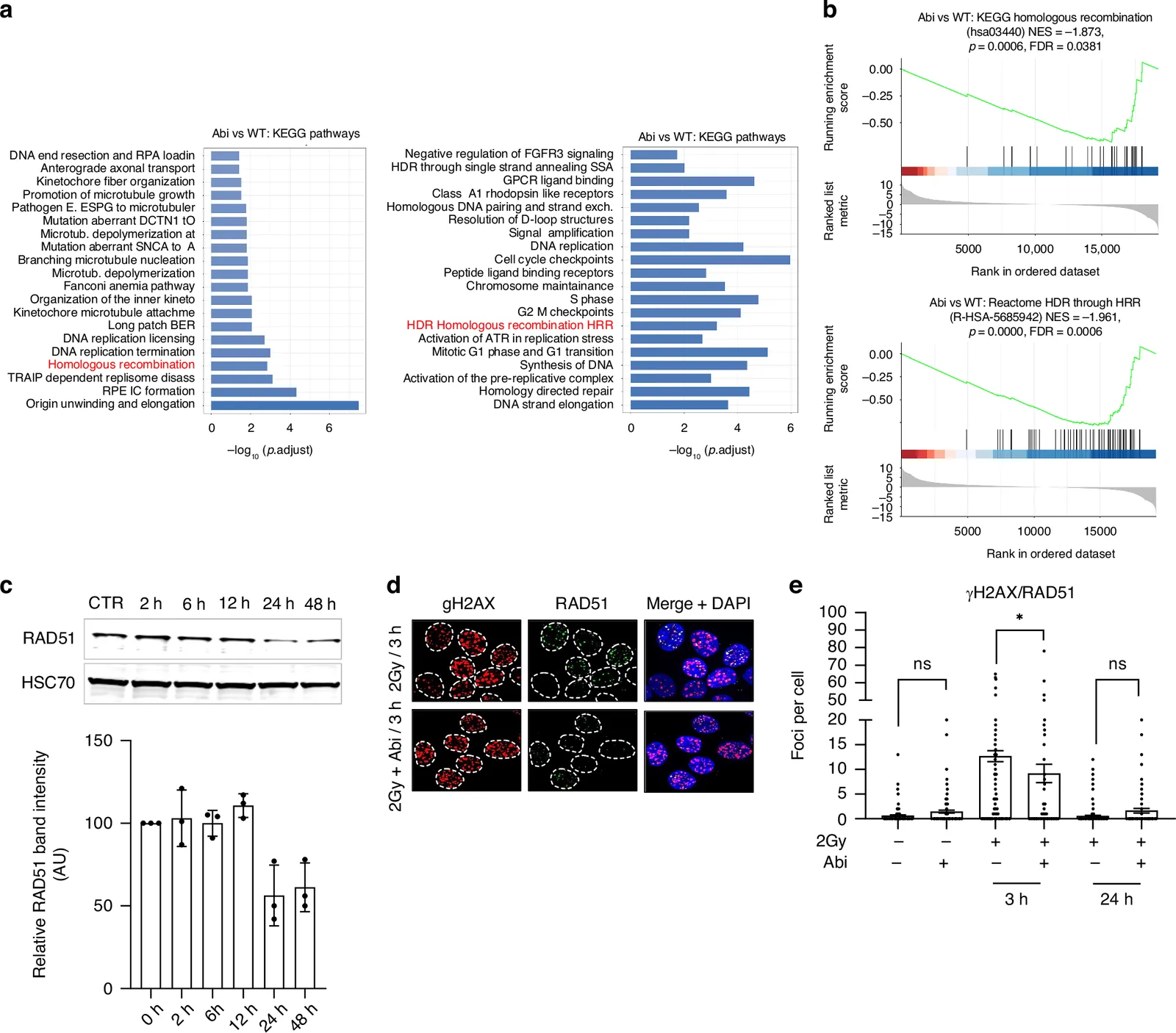
Abiraterone treatment impairs homologous recombination by downregulating RAD51 expression. **a** Pathway enrichment analysis of differentially expressed genes in abiraterone‑treated versus control LNCaP cells using KEGG (left) and Reactome (right) databases. Bars depict significantly downregulated pathways ranked by adjusted P value (−log10 q‑value). Homologous recombination (HR)–related pathways are highlighted in red. **b** Gene set enrichment analysis (GSEA) plots showing negative normalized enrichment scores (NES) for KEGG HR (left) and Reactome “HDR through HRR” (right) gene sets in abiraterone‑treated cells. **c** Upper panel: Western blot showing RAD51 protein expression in LNCaP cells treated with 5 µM abiraterone at the indicated time points. HSC70 was used as a loading control. Lower panel: Quantification of RAD51 band intensities, shown are means ± SD of three independent blots. **d** Representative immunofluorescence images showing γH2AX (red) and RAD51 (green) foci at 3 h and 24 h postirradiation with 2 Gy. DAPI (blue) counterstaining was used to visualize the nuclei. **e** Quantification of γH2AX/RAD51 foci numbers at 3 h and 24 h postirradiation with 2 Gy in LNCaP cells treated with 5 µM abiraterone (Abi) or without (CTR). The data are presented as the means ± SEMs from at least three independent experiments. Statistical significance was determined using an unpaired Mann-Whitney t-test. *p* values: * < 0.05, ns: not significant.

Previous studies have provided insights into how ARPIs, such as abiraterone, interact with DNA repair mechanisms and HRR pathways [10, 36–39]. For example, Li et al. reported that combining ARPIs with olaparib significantly impaired HRR efficiency [37]. To further explore this relationship, we performed RNA-Seq analysis to assess the effect of abiraterone treatment on the transcriptomic profile of androgen-sensitive LNCaP cells. GSEA based on MSigDB revealed downregulation of several DNA repair hallmark gene sets (Fig. S2C-F). Pathway enrichment analysis using KEGG and Reactome, but notably not GO, revealed HR among the most significantly downregulated pathways in abiraterone‑treated cells (Figs. 2a and S2A). Consistently, GSEA based on KEGG, Reactome, and interestingly GO gene sets showed significantly negative normalized enrichment scores for HR‑related pathways, indicating coordinated transcriptional suppression of HR genes upon abiraterone treatment (Figs. 2b and S2B). Among these genes, the key HR factor RAD51 was markedly reduced. Immunoblotting confirmed a time-dependent decrease in RAD51 protein levels following abiraterone exposure (Fig. 2c), suggesting functional HRR deficiency in treated cells. To address this issue, we performed double immunofluorescence staining to monitor γH2AX and RAD51 foci formation and resolution after 2 Gy irradiation, in the presence or absence of abiraterone. Quantitative analyses clearly demonstrate that abiraterone pretreatment significantly increases γH2AX foci numbers versus untreated controls (*p* < 0.01), confirming DSB repair deficiency. Conversely, RAD51 foci are profoundly reduced (*p* < 0.001) (Fig. S3A, B). Of note, while RAD51 expression was decreased, no change in H2AX or 53BP1 protein levels was detected upon exposure to Abi (Fig. S3C). Colocalization analysis further revealed 30% reduced γH2AX/RAD51 loading at 3 h (*p* = 0.017) (Fig. 2d and e). These findings confirm that abiraterone disrupts HR repair by reducing RAD51 expression.

### Abiraterone reduces the repair capacity of ionizing radiation-induced DSBs in fresh PCa ex vivo tissue slice cultures

**Fig. 3:**
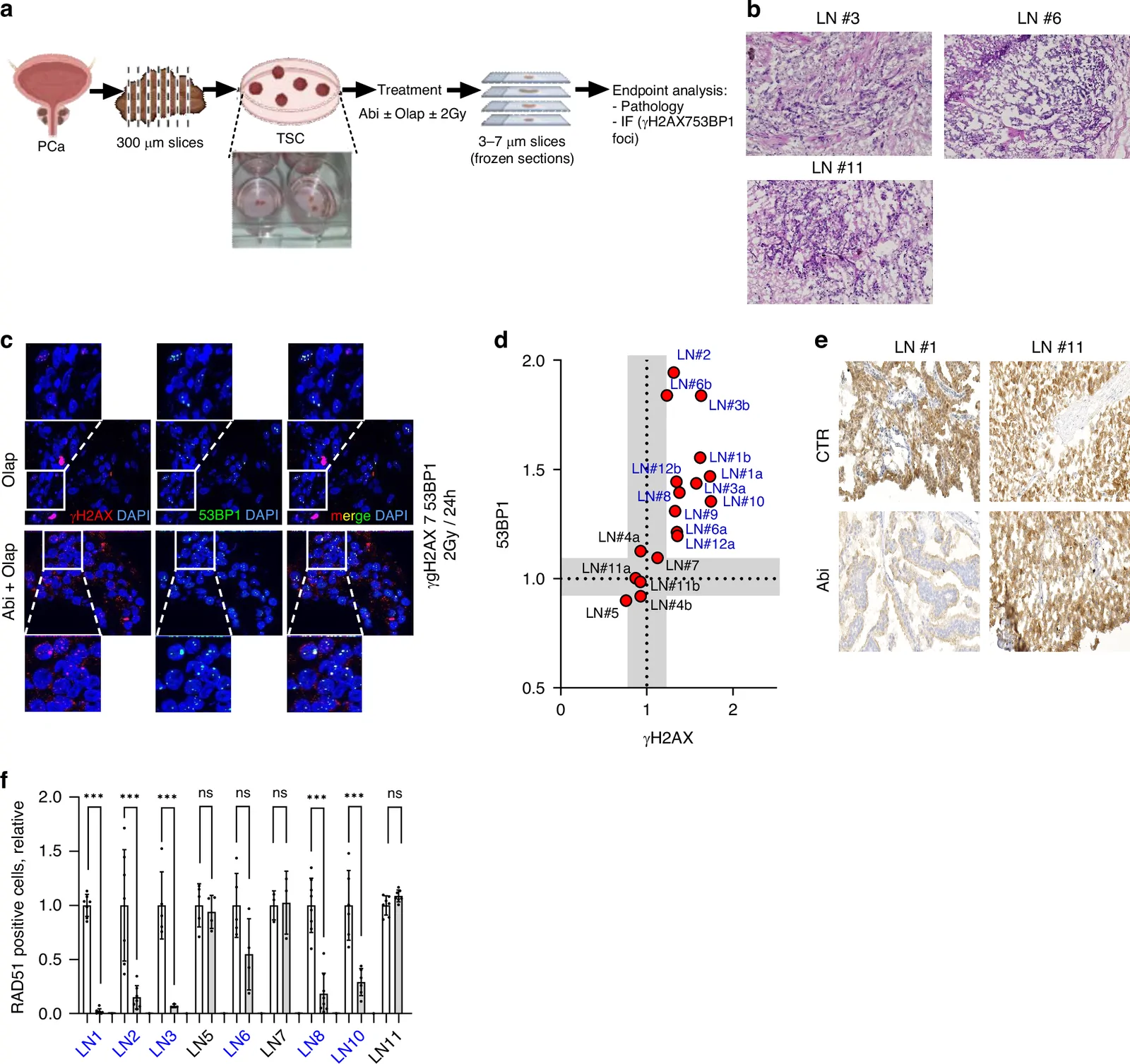
Ex vivo tissue slice cultures demonstrated that combined abiraterone and olaparib treatment impaired the repair of IR-induced DSBs. **a** Schematic diagram of the ex vivo tissue slice culture (TSC) setup. **b** Representative images of H&E staining of lymph node–metastasized tumor slice cultures. **c** Representative immunofluorescence images of TSCs from patient LN #9 pretreated with either 5 µM abiraterone alone (Abi) or in combination with 1 µM olaparib (Abi+Olap) before irradiation with 2 Gy, showing γH2AX (red) and 53BP1 (green) foci at 24 h postirradiation with 2 Gy. DAPI (blue) counterstain was used to visualize the nuclei. **d** Plot showing the correlation between the enhancement ratio (ER) of abiraterone combined with olaparib (Abi+Olap ER) vs Abi+IR and the residual γH2AX (X-axis) and 53BP1 (Y-axis) foci at 24 h after 2 Gy. ER was calculated as the relative increase in mean γH2AX and 53BP1 foci counts individually assessed from ≥3 independent experiments, as previously described [24]. **e** Representative IHC images of RAD51 staining in TSCs from responder (LN1) and non-responder (LN11) patients under control (CTR) or abiraterone (Abi)-treated conditions. **f** Quantitative analysis of relative RAD51-positive cells (normalized to CTR). Shown are mean ± SD of at least six images. Statistical significance was determined using an unpaired Mann-Whitney t-test, ***p < 0.001. ns: not significant.

Ionizing radiation (IR) kills cells by inducing various types of DNA damage, with DSBs being the most toxic [40]. The efficiency of DSB repair is correlated with the sensitivity of cells to IR [40]. Previously, we developed a robust preclinical ex vivo PCa tumor tumor slice culture model that allows monitoring of DSB repair in fresh tumor tissue (Fig. 3a, [24]). Here, we sought to use this ex vivo system to investigate the cytotoxic effects of the combination of abiraterone and olaparib on the repair of IR-induced DSBs. To that end, tissue slice cultures (TSCs) were established from 19 lymph node metastatic PCa patients who underwent PSMA-guided lymph node dissection at Martini-Klinik in Hamburg, Germany. H&E-stained cryosections from each sample were examined by digital pathology for tumor cell content, confirming PCa histology in all cultured lymph node metastasis slices (representative images Fig.3b).The viability and appropriate oxygenation of established TSCs were routinely confirmed by monitoring EdU^+^ cells and performing pimonidazole staining as previously described [24]. Notably, the HRR status of these patients is unknown. These TSCs were initially treated with 5 µM abiraterone for 22 h, after which 1 µM olaparib was added for an additional 2 h before irradiation with 2 Gy. We assessed the treatment effect by monitoring the number of residual DSB markers γH2AX and 53BP1 at 1 h and 24 h post-IR. Previously, we reported that abiraterone inhibits the repair of IR-induced DSBs [13]. In this study, we confirmed this finding and, more importantly, demonstrated that the combination of abiraterone and olaparib more effectively inhibited the repair of IR-induced DSBs, as indicated by a significant increase in residual DSB foci with the combined treatment (Figs. 3c and S4). To emphasize this point, we measured the enhancement ratio (ER) of residual γH2AX as well as 53BP1 foci for the combined treatment compared with abiraterone alone with IR, as previously described [24]. Briefly, ER was defined as the fold change in the means of γH2AX and 53BP1 foci, individually assessed across all TSCs from at least three independent experiments. As shown in Fig. 3d, 12 out of 18 TSCs presented increased enhancement ratios (Table S1). To validate HR inhibition and corroborate prior RAD51 suppression data (Fig. 2), we assessed RAD51 expression via IHC in TSCs from responders versus non-responders post-abiraterone (Fig. 3e). Strikingly, RAD51 remained unchanged in non-responder TSCs (LN5, LN7, LN11) but was potently downregulated in responders (LN1: 98%, LN2: 85%, LN3: 93%, LN6: 46%, LN8: 82%, LN10: 70%) Although the responder TSC LN5 did not exhibit a statistically significant decrease in RAD51 expression after abiraterone treatment, a borderline reduction was also noted (7%,) (Fig. 3f). Collectively, these findings demonstrate the superior efficacy of combination therapy driven by pharmacological BRCAness induction.

### The combination of abiraterone and olaparib is more toxic than a single treatment in metastatic PCa organoids

**Fig. 4:**
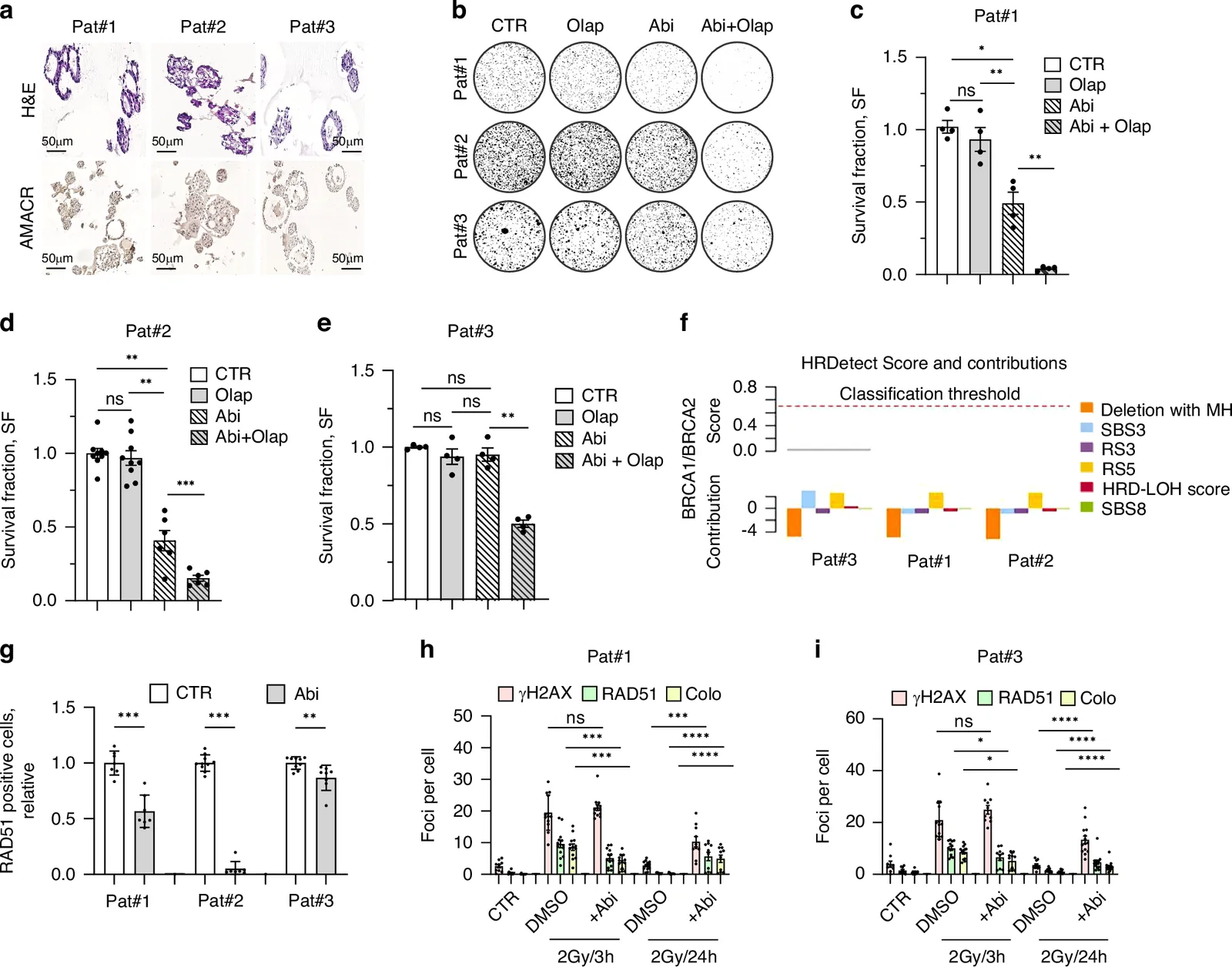
Combined treatment with abiraterone and olaparib results in a more significant decrease in the survival of metastatic patient-derived organoid cultures. **a** Representative histopathological images of H&E and AMACR staining of organoids derived from the indicated mPCa patients. **b** Representative images of organoids from the indicated patients stained with MTT, showing the effects of olaparib (Olap), abiraterone (Abi), and their combination (Abi+Olap) compared with those of the untreated control (CTR). **c**–**e** Quantification of the experiment in (**b**), showing survival fractions measured by 3D CFA for organoids derived from mPCa patients (**c**) Pat#1, (**d**) Pat#2, and (**e**) Pat#3. The data represent the means ± SEMs from three independent experiments conducted for each organoid line. Statistical significance was determined using an unpaired Mann-Whitney t-test, *p < 0.05, **p < 0.01, ***p < 0.001. ns: not significant. **f** Contributions of six mutational signatures to the HRD/BRCAness HRDetect probability scores for the mPDO samples, estimated based on the HRDetect Workflow. None of the samples was classified as HRD based on a BRCAness probability score ≥ 0.7, indicated by the red line. Weighted contributions are shown for microhomology-mediated deletions, single-base substitution signatures (SBS) 3 and 8, structural rearrangement signatures (RSs) 3 and 5, and the loss of heterozygosity (LOH) score. All features were calculated based on somatic variants identified across the whole genomes, including insertions/deletions, single nucleotide variants, structural variants and copy number variants. **g** Quantification of RAD51-positive cells in the three mPDOS samples comparing abiraterone-treated and untreated conditions using the QuPath software platform (v0.6.0). **h**, **i** Quantification of individual or colocalized γH2AX and RAD51 foci in PDOs from (**h**) Pat#1 and (**i**) Pat#3 after the indicated treatments. Statistical significance was determined using an unpaired Mann-Whitney t-test, *p < 0.05, **p < 0.01, ***p < 0.001, ****p < 0.0001. ns: not significant.

PDOs offer a key advantage in preclinical models by enabling the analysis of drug effects on clonogenic survival. We created PDOs from three mPCa patients: two with lymph node metastasis, one with no castration resistance (Pat#1) and one with CR features (Pat#2), and one bone metastatic patient with castration resistance features (Pat#3). Clinical and pathologic characteristics of three PCa patients are shown in Table S2. The mPDOs confirmed their prostatic origin, displaying strong AMCAR staining (Fig. 4a). We examined the effect of a combined treatment regimen on the survival of these mPDOs (Fig. 4b). The PDOs were treated simultaneously with 5 µM abiraterone and 1 µM olaparib. Clonogenic survival was quantified in 3D settings via a colony-forming assay (CFA), as previously described [25]. MTT staining was used to visualize and count living cells within the organoids before harvesting. Olaparib alone did not affect the survival of either PDO. In contrast, abiraterone reduced the survival of PDOs derived from LN-metastatic patients (Pat#1 and Pat#2) by approximately 50% (Fig. 4c and d) but had no effect on the survival of PDOs derived from bone-metastatic patients (Pat#3) (Fig. 4e). Importantly, the combined treatment exhibited dramatic cytotoxicity in all the PDOs ( > 90% in Pat#1 and Pat#2 PDOs and approximately 50% in Pat#3 PDOs) compared with the single treatment (Fig. 4c-e). WGS sequencing of the tested organoids revealed single base substitution (SBS) mutational signatures characteristic of microsatellite instability (MSI) but no alterations linked to intrinsic HR defects, as SBS3 and RS3 signatures showed negligible contributions across all PDOs (Fig. S5).

HRDetect analysis, which assesses HR deficiencies on the basis of mutational patterns [41], revealed no HR-deficient profile with HRDetect scores below the 0.7 classification threshold for all PDOs (Fig. 4f). Strikingly, RAD51 IHC staining demonstrated downregulation upon abiraterone treatment in PCa PDOs (44% in Pat#1, 95% in Pat#2, and 14% in Pat#3) (Figs. S6A and 4g), which was further confirmed by RAD51 protein blotting in the corresponding mPDOs (Fig. S6B). Confirming functional impairment, PDOs from Pat#1 and Pat#3 displayed markedly reduced IR-induced DSB repair efficiency post-Abi treatment (Fig. S6C&D). Most compellingly, RAD51 focus formation at DSB sites was profoundly diminished across models (Fig. 4h, i), solidifying pharmacological BRCAness induction independent of intrinsic HR defects and directly accounting for the observed cytotoxicity.

### Like abiraterone, both apalutamide and enzalutamide reduce HRR pathway gene expression

**Fig. 5:**
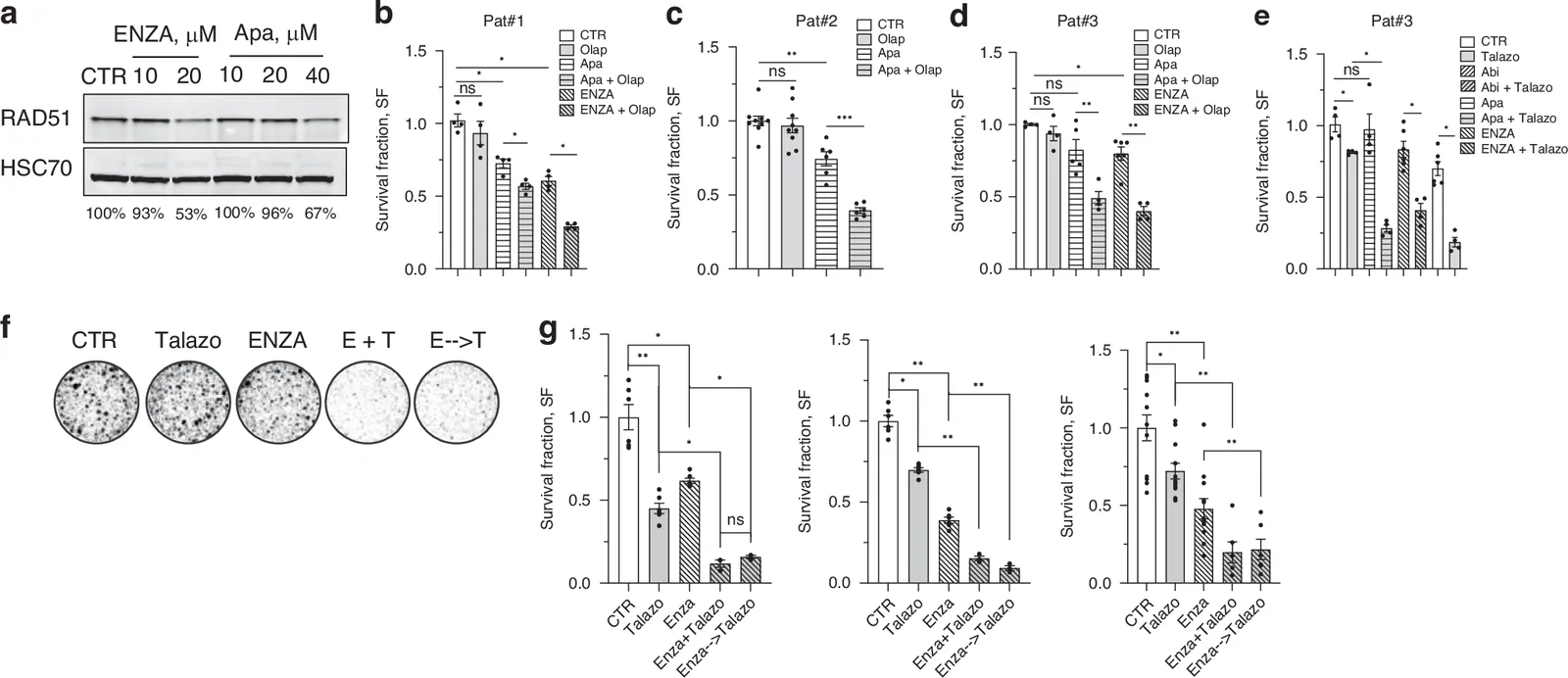
Enhanced cytotoxicity in metastatic patient-derived organoids with combined PARPi and ARPI treatment via simultaneous or sequential administration. **a** Western blot analysis of LNCaP cells treated with enzalutamide (Enza; 10, 20 µM) or apalutamide (Apa; 10, 20, 40 µM), showing dose-dependent downregulation of RAD51 protein levels (HSC70 loading control). Relative band intensities (% of CTR) are indicated below. Effects of the indicated treatments on the survival of organoids from **m**PCa patients: **b** Pat#1, **c** Pat#2, and **d** Pat#3. **e** Survival fractions in organoids from patient Pat#3 after the indicated treatments. Like olaparib, talazoparib (Talazo) induced greater cytotoxicity in survival fractions when combined with abiraterone (Abi), enzalutamide (ENZA) or apalutamide (Apa). The data represent the means ± SEMs from three independent experiments conducted for each patient-derived organoid. The CTR and Olap survival values of Pat#1, #2 and #3 are taken from Fig. 4. **f** Talazoparib and enzalutamide were combined and administered either simultaneously (Enza+Talazo) or sequentially (Enza→Talazo), and **g** the effects on the survival of organoids from mPCa patients Pat#1, Pat#2, and Pat#3. were measured via a colony formation assay. The data represent the means ± SEMs from three independent experiments conducted for each PDO. Significance in all experiments in Fig. 5 was determined by unpaired Mann–Whitney t-test (*P < 0.05, **P < 0.01, ***P < 0.001); ns, not significant.

The data above demonstrate that ARPI abiraterone induces a BRCAness phenotype, increasing the sensitivity of cells to olaparib. To determine whether this effect is common among ARPIs, LNCaP cells were treated with the androgen receptor inhibitor apalutamide (40 µM) or enzalutamide (20 µM) for 24 h, followed by RNA-Seq analysis to assess transcriptome changes. Pathway enrichment analysis of these treatments revealed a common downregulation of DNA repair-related pathways, including HRR gene sets in GO, KEGG, and Reactome analyses as well as negative normalized enrichment scores (NES) in GSEA plots for HR-using LNCaP cells (Figs. S7A&B and S8A&B). Western blot interestingly recapitulated the decreased levels of RAD51 protein also upon Apa (67% at 40 µM) or Enza (53% at 20 µM) treatment in a dose-dependent manner (Fig. 5a). This suggests the induction of BRCAness, which is further supported by the more pronounced inhibitory effects on the survival of PDOs from LN-metastatic (Pat#1 and Pat#2) or bone metastatic (Pat#3) PCa patients (Fig. 5b-d). Interestingly, similar results were also confirmed when the PARPi talazoparib (100 nM) was used instead of olaparib (Fig. 5e), further supporting the superiority of combining ARPIs with PARPis over individual treatments. In the above experiments, we used simultaneous treatment with both drugs for the combined treatment, which improved the treatment effect over the individual treatments of either drug alone. Next, we investigated whether sequential administration of the combined treatment would be beneficial by comparing the effects of simultaneous and sequential treatments on the survival of the three mPDOs. To this end, talazoparib was used as a PARPi, and enzalutamide was used as an ARPI. PDOs from Pat#1, Pat#2, and Pat#3 patients were treated with 20 µM enzalutamide, 100 nM talazoparib, or enzalutamide followed by talazoparib (24 h apart), and survival was assessed via 3D CFA. Interestingly, both treatment strategies had similar effects on the survival of the indicated mPDOs (Fig. 5f&g).

### Olaparib treatment reduces prometastatic protein levels in mPCa organoids

**Fig. 6:**
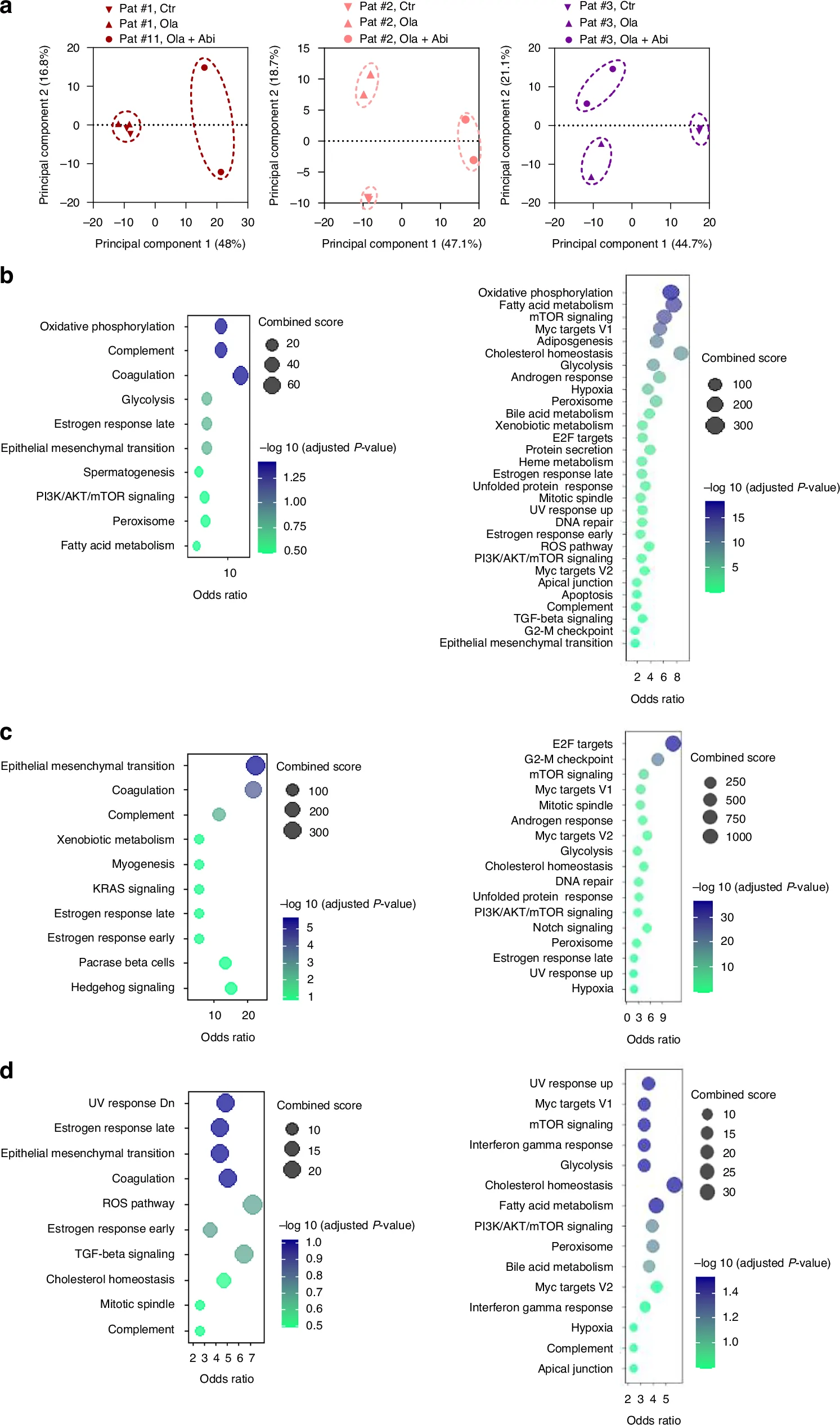
Proteomic profiles of PDOs generated from mPCa patients under different treatment conditions. **a** PCA scatter plots of all PDOs from three mPCa patients (Pat#1: dark brown, Pat#2: pink, and Pat#3: purple), highlighting the indicated treatment conditions. **b**–**d** Left panels: Pathway enrichment analysis based on significantly low-abundance proteins (fold change >1.5) in (**b**) Pat#1 (**c**) Pat#2 and (**d**) Pat#3 mPDOs following olaparib treatment compared with untreated controls (44 proteins in Pat#1, 41 in Pat#2, and 123 in Pat#3). Right panels: Pathway enrichment analysis in the mPDOs established from (**b**) Pat#1, (**c**) Pat#2, and (**d**) Pat#3, based on the significantly low abundant proteins following combined treatment with olaparib and abiraterone compared to olaparib alone (865 proteins in Pat#1, 877 in Pat#2, and 264 in Pat#3; *p* < 0.05, Table S3). Significantly enriched pathways are presented as bubble plots. The odds ratio on the x-axis represents the overrepresentation of genes in each pathway, whereas the y-axis represents the enriched terms. The bubble color corresponds to the adjusted *p* value (*q* < 0.05), and a higher log10 value (adjusted P value) reflects greater statistical significance. The combined scores, reflecting both the *p* value and odds ratio, are reflected by bubble size, with larger bubbles indicating greater statistical significance and enrichment.

In the PROpel study, patients had not received prior treatment for metastatic disease, yet they benefited from the combination of abiraterone and olaparib. However, it remains unclear whether this combination specifically targets metastases or simply exerts cytotoxic effects without affecting metastatic progression. Previously, we demonstrated that olaparib inhibits the metastatic potential of PCa cells, independent of its killing activity [25]. These findings suggest that combining PARPis with ARPIs may represent a dual-hit strategy: on the one hand, PARPis eliminate cancer cells following ARPI-induced pharmacological BRCAness; on the other hand, they may suppress the metastatic potential of these cells. In this study, we aimed to explore this effect in more detail by investigating the effect of olaparib on the proteomic profile of treated cells. To this end, PDOs established from mPCa patients were treated with 1 µM olaparib, with or without 5 µM abiraterone, for 24 h. Subsequently, bottom-up quantitative proteomics with LC‒MS/MS was conducted to compare their proteomic profiles. A total of 9271 proteins across all the studied mPDOs were identified (Table S3). Principal component analysis (PCA) revealed clear differences among the three mPDOs and high similarity between the biological replicates within each sample group (Fig. S9A). The effects of olaparib, alone or in combination with abiraterone, on individual organoid lines were visualized via PCA (Fig. 6a). Pathway enrichment analysis on the basis of the significantly low-abundance proteins with a fold change >1.5 revealed that several proteins downregulated following olaparib treatment are key regulators of metastasis, particularly those involved in epithelial‒mesenchymal transition (EMT) (Fig. 6 b-d, Table S4). These findings align with our previous study, which demonstrated olaparib’s inhibitory effect on PCa metastatic potential through reduced cell migration [25]. Consistent with our prior observations that abiraterone reduces the expression of DNA repair genes and induces a BRCAness phenotype, a comparison of protein expression profiles between the combined treatment and either untreated or olaparib-treated mPDOs revealed consistent downregulation of DDR and HRR genes, including RAD51 (Table S5 and Fig. S9B). Furthermore, when the combination was directly compared with olaparib alone, the expression of DDR- and DNA repair–related proteins remained significantly reduced (Fig. 6b-d, right panels).

Collectively, these findings (i) further support the induction of a BRCAness-like phenotype by abiraterone treatment and (ii) suggest that olaparib may suppress metastasis by downregulating prometastatic gene expression.

## Discussion

The current study aimed to explore the biological mechanisms underlying the findings of a clinical trial and revealed that combining PARPis with ARPIs improved mPCa patient outcomes. The phase III PROpel trial demonstrated that olaparib plus abiraterone significantly improved radiographic progression-free survival in mCRPC patients and clinically meaningful improvement in OS. Additionally, the TALAPRO-2 study showed similar efficacy to talazoparib and enzalutamide. While preevaluation of HR status via next-generation sequencing (NGS) is an important step for the clinical use of PARPis, both the PROpel and TALAPRO-2 trials interestingly reported that the combination of ARPIs with PARPis is effective for patients with or without HRR defects. Despite these findings, the exact mechanisms driving the benefits in both patient groups remain unclear. Our study demonstrates that ARPIs induce a BRCAness-like functional state in PCa, which is characterized by (i) reduced RAD51 protein expression across all models tested, (ii) impaired RAD51 recruitment to DSBs despite (iii) persistent γH2AX foci, indicating DSB repair failure, and (iv) olaparib hypersensitivity across models. Unlike classical BRCAness defined by underlying germline or somatic DDR mutations, this pharmacologically-induced phenotype is driven by a state of ‘’repair exhaustion”, characterized by the broad suppression of canonical DNA repair pathways. This contradicts with the compensatory upregulation of repair machinery often observed in basal high-BRCAness tumors [42]. This induced repair deficiency defines a unique functional vulnerability, providing a robust rationale for exploiting this pharmacologically sensitized state to enhance the efficacy of DNA-damaging cancer therapies. Consequently, this pharmacologically induced HRR deficiency sensitizes PCa models to PARP inhibition, independent of pre-existing genetic HRR defects, supporting the rationale for combining ARPI with PARPi, even for patients with an HRR-wild-type background. Our RNA and proteomic analyses revealed that treatment with abiraterone, enzalutamide, or apalutamide reduces the expression of several DDR and DNA repair genes, including the HRR protein RAD51, leading to BRCAness phenotype induction, as evidenced by impaired RAD51 loading at IR-induced DSB sites. Proteomic analysis further reveals that ARPI+PARPi combincation downregulates EMT-related proteins, probably extending the BRCAness concept beyond DNA repair to suppress metastasis-associated pathways. This offers a novel therapeutic vulnerability that may explain the enhanced clinical efficacy of this regimen in overcoming PCa resistance. Consequently, abiraterone+olaparib combination therapy demonstrated superior efficacy compared with single agents across hormone-sensitive LNCaP and castration-resistant DU145/22RV1 cells, validated in patient-derived models, including lymph node mHSPC and bone mCRPC TSCs/PDOs. Apalutamide and enzalutamide similarly induced BRCAness via RAD51 downregulation, yielding enhanced cytotoxicity with olaparib. Talazoparib+ARPI combinations produced comparable synergy. Notably, DDR and/or HRR status in our pre-clinical models (TSC and PDOs) was unknown, reflecting our hypothesis that ARPIs+PARPi efficacy operates independently of DDR alterations. With BRCA1/2 mutation rates ~5-8% in mPCa, the observed >60% response rate in the pre-clinical patient-derived models far exceeds this prevalence. Moreover, PARPi monotherapy showed minimal activity, confirming that ARPIs pretreatment induces functional HRR deficiency rather than exploiting pre-existing defects. Accordingly, a previous study demonstrated that treatment with enzalutamide decreases the levels of key HR proteins, such as RAD51, leading to BRCAness and rendering CRPC cells sensitive to olaparib [37].

An established interplay between AR signaling and DNA repair has been documented [10, 43]. We previously demonstrated that ARPI treatment impairs DNA DSB repair in PCa cell lines [13]. AR directly binds regulatory elements within several DNA repair genes, including those involved in HRR, to modulate their expression and sustain HRR activity [44]. Following ADT, cells become more dependent on PARP-mediated repair, making them more sensitive to PARP inhibition [44]. Several studies have shown that AR signaling upregulates DDR genes [10, 43, 45], some of which are associated with PCa metastasis [45]. In the TALAPRO-2 trial, TMPRSS2-ERG and RB1 were identified as predictive biomarkers for the efficacy of combining talazoparib and enzalutamide in HRR-negative patients [46]. These genes were previously linked to a BRCAness phenotype, as ERG overexpression via TMPRSS2-ERG fusion was shown to induce a BRCAness phenotype [47], and RB1 loss was found to impedes DNA DSB ends resection and HRR [48]. In both instances, the induced BRCAness phenotype renders cells harboring these alterations particularly sensitive to PARPi monotherapy. Analysis of HRDetect scores suggested that the treatment response observed in the current study was not driven by a preexisting BRCAness phenotype in the models. Furthermore, the combination of a PARPi with an ARPI consistently produced greater cytotoxicity than either agent alone did, even in models where PARPi monotherapy had little to no effect.

Another key question addressed in the current study is whether the sequence of drug administration affects the efficacy of combining ARPI with PARPis. We investigated two strategies: pretreatment with ARPI followed by treatment with a PARPi and simultaneous administration of both drugs. Our results show that both approaches effectively inhibit tumor survival more effectively than either drug alone does. Recently, preliminary data from the BRCAAway trial, presented by Hussien et al. at ASCO GU 2024, demonstrated that the simultaneous combination of abiraterone and olaparib led to improved survival compared with sequential therapy [49]. While this seems to contradict our findings, it is important to note that in the BRCAAway trial, sequential treatment began only after disease progression following the first treatment, which differs from the 24 h separation between the two treatments in our study. This difference in study design may help explain the contrasting results between the BRCAAway trial and our current study. In our preclinical investigation, short-interval sequential administration of an ARPI followed by a PARPi demonstrated an effect comparable to that of concomitant treatment. This finding underscores the capacity of AR-targeted agents to induce a BRCAness phenotype. However, these results cannot be directly extrapolated to clinical practice, where patients typically receive ARPIs for a prolonged period prior to the initiation of PARP inhibition. The impact of this temporal factor on the extent and durability of BRCAness induction remains unclear. Notably, both the BRCAAway trial and a subgroup analysis of the MAGNITUDE study, which evaluated the combination of niraparib and abiraterone in HRR-positive patients, indicated a diminished therapeutic effect when PARPis were administered sequentially or with temporal delay. Although our study demonstrates significant AR-dependent DNA repair vulnerabilities in PCa, we recognize critical limitations in our experimental approach. Specifically, while the LNCaP cell line is considered a robust, androgen-sensitive platform for probing AR pathway inhibition, this cell line fails to recapitulate the molecular heterogeneity of advanced disease. Likewise, the modest size of our PDO cohort limits the broader applicability of our observations. Nonetheless, these findings offer strong proof-of-concept for targeting these vulnerabilities, meriting further exploration in heterogeneous preclinical systems and expanded.

## Supplementary information

Supplementary Figures

Supplementary Table S1

Supplemetary Table S2

Supplementary Table S3

Supplementary Table S4

Supplementary Table S5

## Data Availability

The data generated in this study are available upon request from the corresponding author. The mass spectrometry proteomics data have been deposited to the ProteomeXchange Consortium via the PRIDE [34] partner repository with the dataset identifier PXD063764.
